# Integrated analysis of mRNA and miRNA expression profiles in livers of Yimeng black pigs with extreme phenotypes for backfat thickness

**DOI:** 10.18632/oncotarget.21918

**Published:** 2017-10-19

**Authors:** Wentong Li, Yalan Yang, Ying Liu, Shuai Liu, Xiuxiu Li, Yingping Wang, Yanmin Zhang, Hui Tang, Rong Zhou, Kui Li

**Affiliations:** ^1^ School of Life Science, Foshan University, Foshan 528231, P.R. China; ^2^ College of Animal Science and Technology, Shandong Agricultural University, Tai’an 271000, P.R. China; ^3^ The State Key Laboratory of Animal Nutrition, Institute of Animal Science, Chinese Academy of Agricultural Sciences, Beijing 100193, P.R. China; ^4^ Shandong Lansi Seeds Industry Co., Ltd., Rizhao 276800, P.R.China

**Keywords:** pig, backfat thickness, liver, RNA-Seq, miRNA-seq

## Abstract

Fat deposition is an important economic trait in farm animals as well as obesity related diseases in humans, and the liver is a central organ involved in regulating lipid synthesis and metabolism in mammals. In this study, the pig liver transcriptome of two groups (H and L) showing differences in backfat thickness were profiled using RNA-Seq and miRNA-Seq to further explore the molecular mechanism of fat deposition. A total of 238 differentially expressed genes (DEGs) and 58 differentially expressed miRNAs were identified between the H and L group. These genes and miRNAs were functionally related to lipid metabolism, including *CYP1A1/2*, *HMGCS2*, *ACSS2*, *UBE2L6*, miR-27a, and miR-31. Functional enrichment analysis revealed that genes associated with oxidative stress might be responsible for fat deposition in pigs. Two miRNA-mRNA interaction networks involved in lipid metabolism were identified, and these provided new insights into the molecular regulation that determines fat content in these pigs. Overall, our study furthers our understanding of the molecular mechanisms involved in fat deposition, and these results may help in the design of selection strategies to improve the quality of pork meat and to combat obesity in humans.

## INTRODUCTION

Pig (*Sus scrofa*) is a primary source of animal protein for human consumption. For pig breeding, fat content is an important consideration and indicator, as it is correlated with meat quality and consumer palatability [1]. Furthermore, pigs served as an important model for the genetic basis of obesity, and this is due to their similarity to humans in terms of genetics, body size, and other physiological and anatomical features [2]. Different selection strategies during pig breeding lead to significant differences in fat content among pigs. The accumulation of fat appears to be a result of excessive fat intake relative to fat oxidation, and the amount of fat deposition is determined by the synthesis and storage of triglycerides, lipid mobilization, and fatty acid oxidation. The liver plays a key role in regulating lipid synthesis and metabolism in mammals, especially in regard to the process of triglyceride synthesis, as it is the central organ involved in *de novo* lipogenesis, gluconeogenesis, and cholesterol metabolism [3, 4], and the process of lipid synthesis in the liver can significantly influence intramuscular fat (IMF) and subcutaneous fat (SAT) deposition in pigs [5].

The molecular mechanism of fat deposition is complex and is affected by many regulatory factors, including mRNAs, non-coding RNAs, and DNA methylation. For example, fat mass and obesity-associated protein (*FTO*) was the first obesity risk gene identified by genome-wide association studies [6, 7], and the *Leptin* gene plays a major role in the regulation of body weight and adipose mass [8, 9]. microRNAs (miRNAs) regulate the process of fat metabolism, mainly by acting on transcription factors to regulate the signaling pathways involved in adipocyte differentiation or by preventing the proliferation of cells in order to promote or inhibit the differentiation of fat cells [10-13]. miR-140 is a facilitator of adipocyte lineage commitment, and it promotes fat deposition via down-regulation of osteopetrosis-associated transmembrane protein 1 (*OSTM1*), which is an anti-adipogenic factor [14]. miR-27 can suppress the terminal differentiation of pre-adipocytes by targeting the adipogenic master genes, *PPARγ* and *prohibitin* [15-17]. Peroxisome proliferator-activated receptor γ (*PPARγ*) is a key transcription factor involved in adipocyte differentiation [18], and *prohibitin* silencing can induce down-regulation of *PPARγ* and reduction of adipogenesis [19]. Other miRNAs, such as miR-17/92 [20], miR-143 [21], and miR-378 [22], have also been shown to play important roles during adipogenesis. However, the molecular mechanism of fat deposition in pigs is still poorly understood.

With the development of high-throughput sequencing technologies, comparative analysis of the liver mRNA transcriptome has been performed to identify candidate genes that affect lipid deposition in pigs [23, 24] and other mammals. An mRNA-miRNA integrative approach has been widely used to study potential interactions between mRNAs and miRNAs based on paired expression profiles, and this approach has identified candidate genes associated with growth and meat quality traits in pigs [25-28]. However, to our knowledge there are as yet no studies integrating the transcriptomes of mRNAs and miRNAs to explore the potential molecular mechanism of fat deposition in pigs with extreme differences in backfat thickness. In this study, we used mRNA and miRNA sequencing to explore the liver expression profile in Yimeng black pigs (an indigenous Chinese pig breed) with extreme differences in backfat thickness to investigate how liver metabolism affects the fat phenotypes of pigs. The purpose of our study was to explore the molecular mechanisms of fat deposition and identify new candidate genes that might be useful for breeding better quality meat in pigs and for combating obesity in humans.

## RESULTS

### Phenotypic variation between extreme groups in backfat thickness

**Figure 1:**
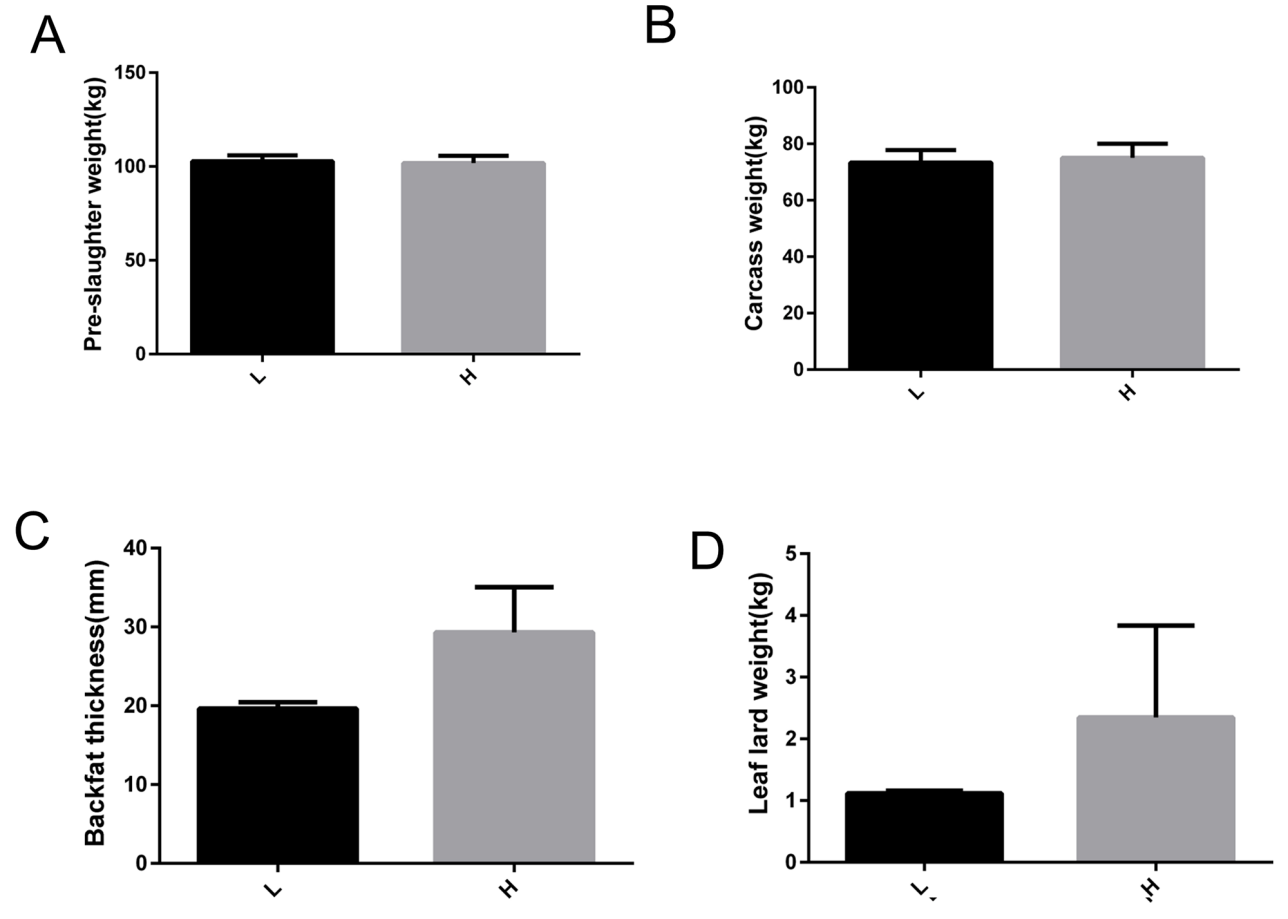
Phenotype information related to fat deposition of Yimeng black pigs with extreme backfat thickness **(A)** Pre-slaughter weight. **(B)** Carcass weight. **(C)** Backfat thickness. **(D)** Leaf lard weight. H, Yimeng black pig group with higher backfat thickness; L, Yimeng black pig group with lower backfat thickness.

Based on backfat thickness, two groups (6 pigs per group) with distinct backfat thicknesses were generated: pigs with higher backfat thickness (H group) and those with lower backfat thickness (L group). The production and carcass traits of these two groups are shown in Table 1. These two groups had no significant differences in body weight (p = 0.398), carcass weight (p = 0.848), leaf lard weight (p = 0.712), and lean percentage (p = 0.393), but they did have significant differences in backfat thickness (p = 0.001) and fat rate (p = 0.039) (Figure 1). Two pigs with high backfat thickness (H group, 29.32 ± 4.06 mm) and two with low backfat thickness (L group, 19.65 ± 0.56 mm) were selected for RNA-seq and miRNA-seq analysis.

**Table 1:** Phenotype information related to fat deposition of Yimeng black pigs

	H	L	*P*-value
Number	6	6	
Body weight (kg)	100.417±1.562	102.167±1.222	0.398
Carcass weight (kg)	74.19±2.355	73.613±1.748	0.848
Slaughter rate (%)	73.836±1.725	72.052±1.454	0.447
Fat rate (%)	24.121±0.677	20.644±1.294	0.039
Lean percentage (%)	52.461±0.928	54.330±1.875	0.393
Backfat thicknesses (mm)	29.819±1.134	21.558±1.351	0.001
Loin muscle area (cm^2^)	17.920±1.083	20.797±3.410	0.440
Leaf lard weight (kg)	1.733±0.152	1.580±0.373	0.712

H, Yimeng black pig group with higher backfat thickness. L, Yimeng black pig group with lower backfat thickness. *P*-value was calculated by t-test.

### RNA sequencing data mapping and annotation

**Figure 2:**
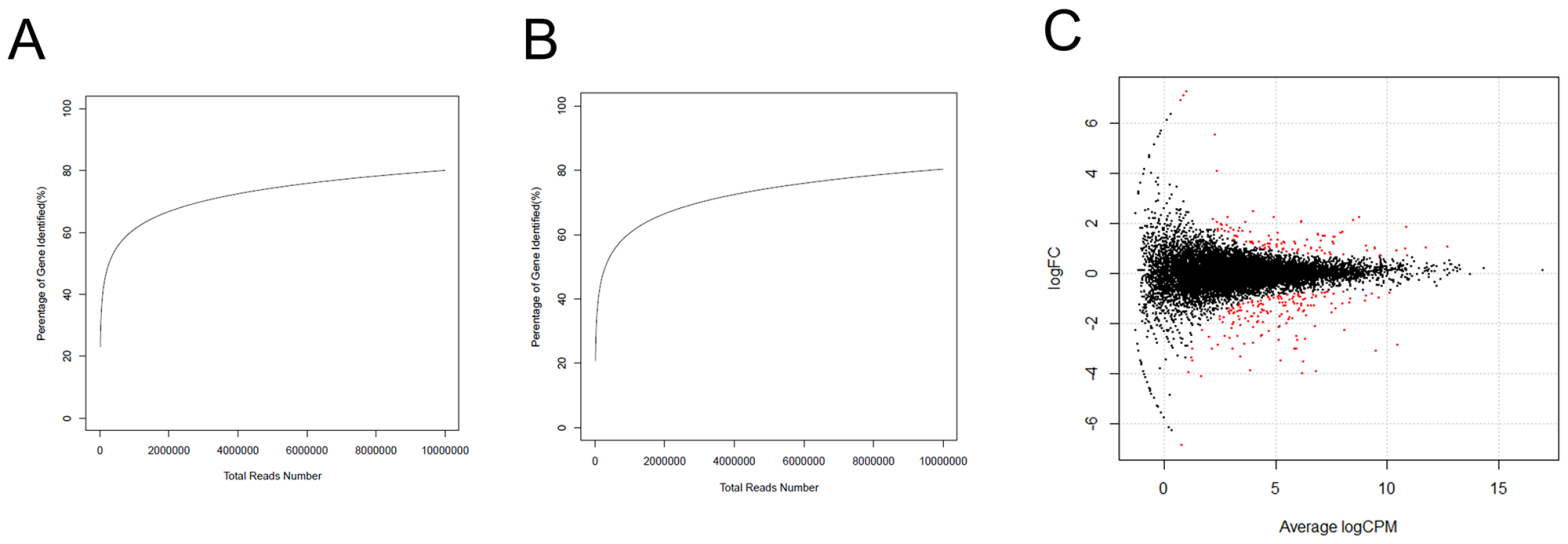
Identification of DEGs between H and L groups **(A)** The test of RNA-Seq saturation in the H group. **(B)** The test of RNA-Seq saturation in the L group. **(C)** Expression scatter plot of the DEGs between H and L groups. Red dot represents a gene with fold change ≥ 1.5 and *P*-value ≤ 0.05. X-axis values are average log2 (counts per million) and y-axis values are log2 (fold change).

Two cDNA libraries of liver tissues from the H and L groups were sequenced. A total of 9,501,761 (H) and 8,993,460 (L) raw reads were obtained from high-throughput sequencing. After removing the adaptors, junk, and low copy reads, 9,481,467 (99.79%) and 8,959,750 (99.63%) clean reads were retained in the H and L groups, respectively. It was observed that 59.75% (H) and 60.40% (L) of the clean reads were mapped to the *Sus scrofa* reference genome (build 10.2). Saturation analysis of the sequencing data showed that the detected genes that were mapped by all clean reads were saturated when the reads counts approached 5 million (Figure 2A-2B). Therefore, the sequencing depth used here was sufficient for transcriptome coverage and subsequent analysis. Additionally, 17,085 and 16,959 expressed genes (RPKM ≥ 0.5) were detected in the H and L groups, respectively. *ALB*, *APOC3*, *COX1*, *COX2*, *COX3*, and *APOE* were the most highly expressed protein coding genes in the liver.

### DEGs between the H and L groups

A total of 238 genes were differentially expressed, with the criteria of at least a 1.5-fold difference and an FDR-value less than 0.05. Of these, 148 were upregulated and 90 were down-regulated in the H group in comparison to the L group (Figure 2C). *SLITRK5* and *RSAD2* were the most upregulated genes, while *MS4A7* and *ABCC8* were the most down-regulated genes in the H group.

### Functional annotation analysis of differentially expressed genes

**Figure 3:**
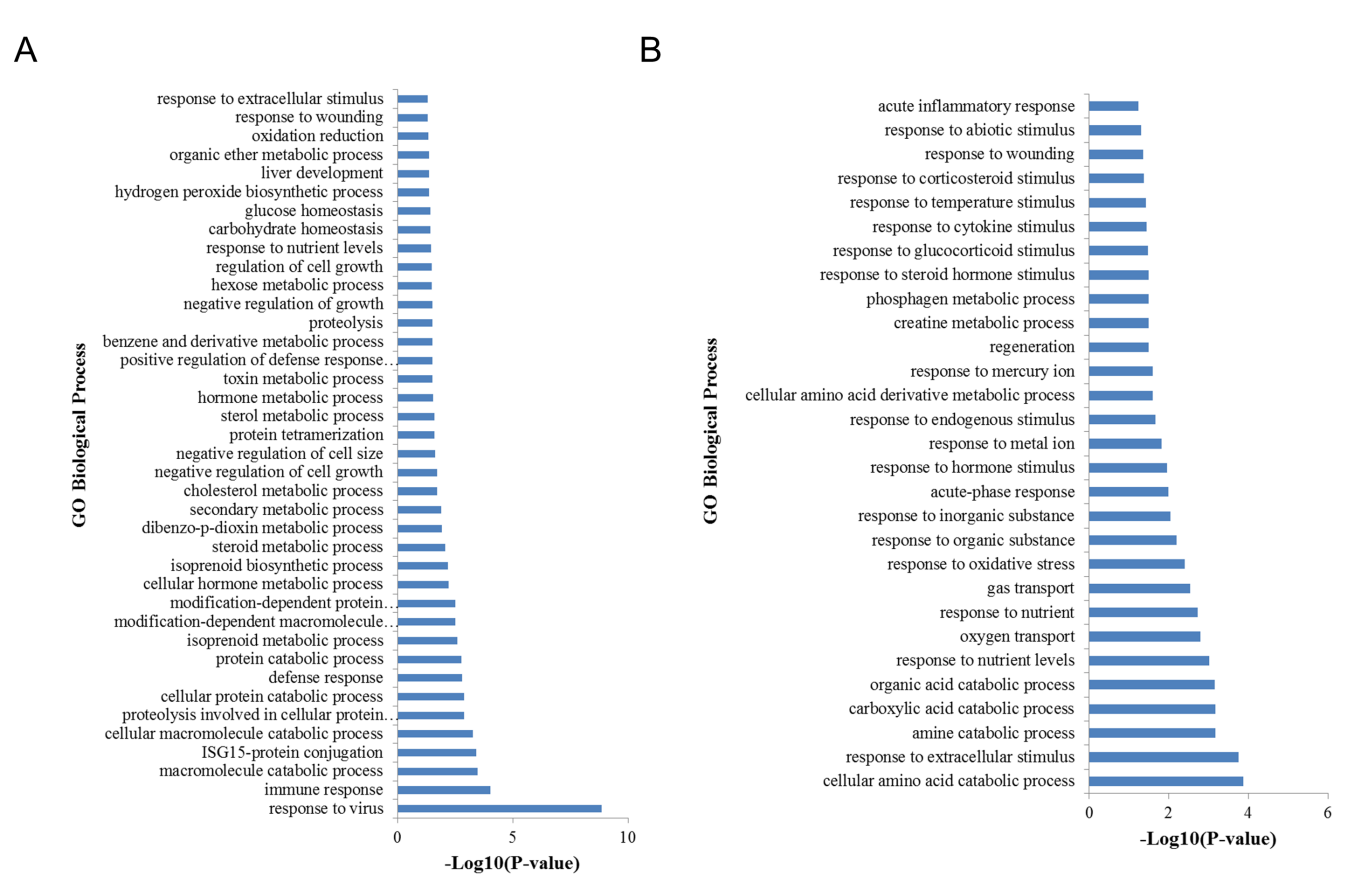
GO biological process analysis of DEGs **(A)** GO biological process analysis of upregulated DEGs. **(B)** GO biological process analysis of down-regulated DEGs when comparing H to L groups.

Gene Ontology (GO) and Kyoto Encyclopedia of Genes and Genomes (KEGG) pathway analyses were performed to investigate the potential functions of the differentially expressed genes. The upregulated genes were significantly associated in 39 GO biological process terms, including immune response, cellular protein catabolic process, cholesterol metabolic process, and oxidation reduction (Figure 3A). KEGG analysis revealed that the upregulated genes were significantly enriched in three pathways, including RIG-I-like receptor signaling pathway, cytosolic DNA-sensing pathway, and retinol metabolism. The down-regulated genes were significantly enriched in 29 GO biological process terms (p < 0.05), including response to extracellular stimulus, oxygen transport, response to oxidative stress, and response to hormone stimulus (Figure 3B). KEGG pathway analysis revealed that these down-regulated DEGs were significantly involved in glycine, serine and threonine metabolism as well as arginine and proline metabolism (p < 0.05).

### Mapping and annotation of miRNA sequencing data

**Figure 4:**
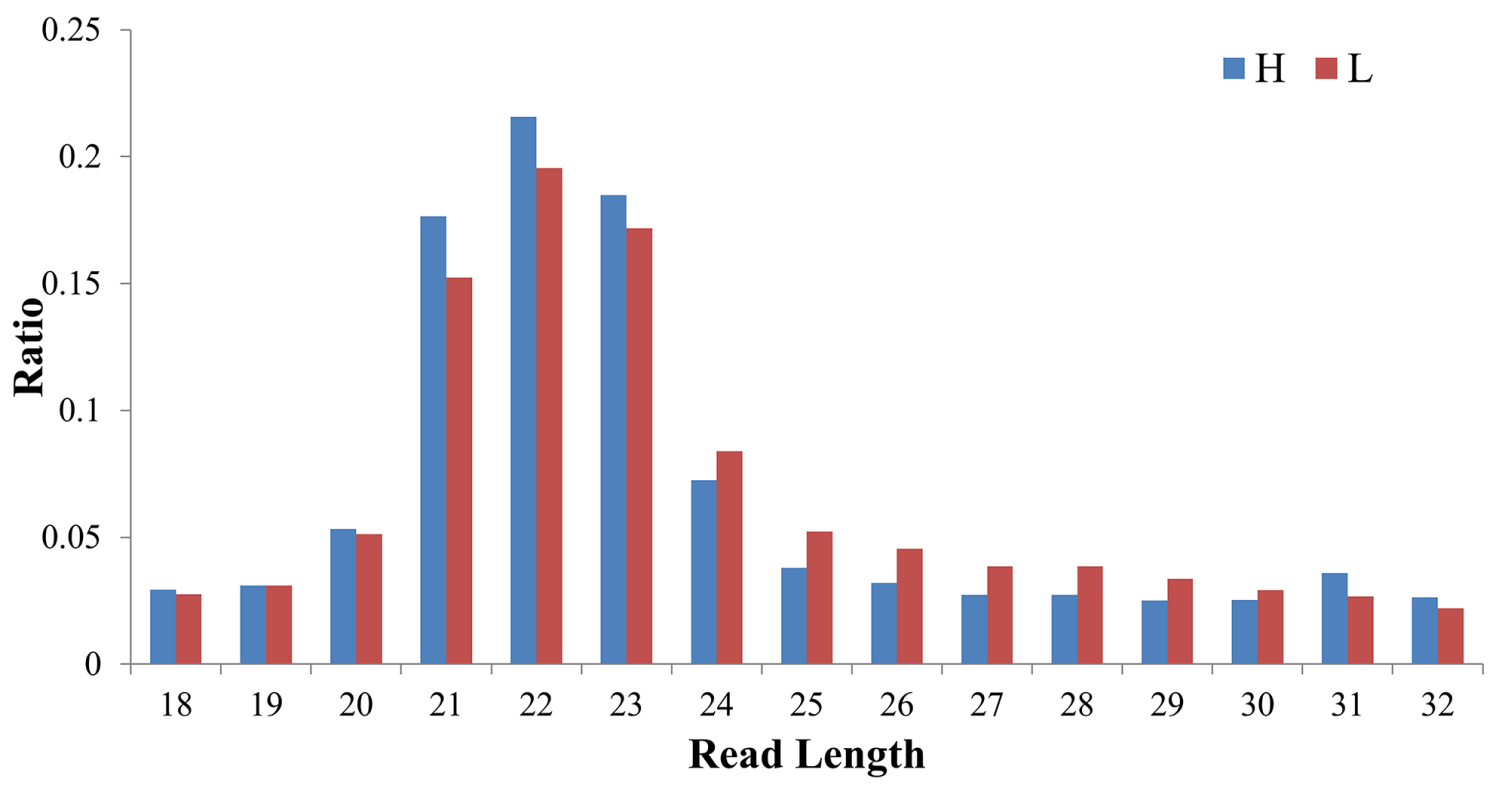
The length distribution of clean reads in the sRNA-seq libraries of H and L groups

Two small-RNA libraries were sequenced from the liver tissues of the H and L groups. After sequencing, a total of 10,688,889 reads were obtained from the H group and 10,781,804 reads from the L group. After removing reads with low quality, the 3′ adapter was trimmed and sequences shorter than 18 nt and longer than 32 nt were discarded. In total, 9,243,990 (H) and 9,347,647 (L) clean reads were obtained, which corresponded to 87.45% and 85.73% of the raw reads from each small RNA (sRNA) library, respectively. The length distribution of clean reads showed that most of the reads were between 21–23 nt in length and read counts of 22 nt were highest (Figure 4). It was observed that 83.5% (H) and 82.3% (L) of the clean reads could be mapped to the porcine reference genome (*Sus scrofa* 10.2). A total of 406 mature miRNAs were identified, and among these a total of 274 known miRNAs that were already annotated in miRbase v21 were expressed. Moreover, 142 novel miRNAs were detected by miRDeep2, of which 100 were miRNAs homologous to human, mouse, rat, or bovine, while 42 were novel miRNAs not homologous to any these four species.

### DE miRNAs between the H group and the L group and pathway analysis

**Figure 5:**
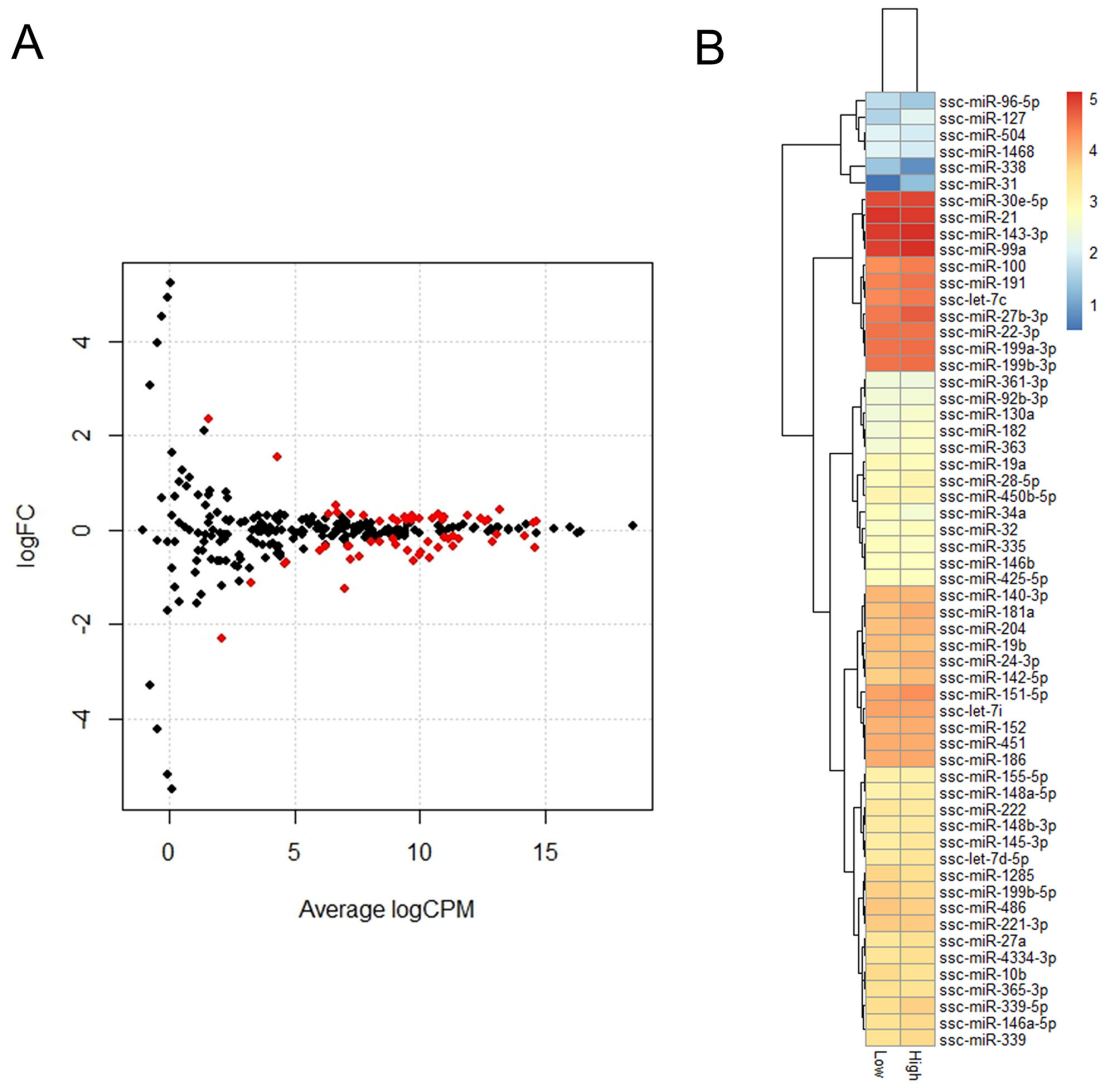
Identification of DEGs between H and L groups **(A)** Expression scatter plot of differentially expressed miRNAs (represented in red) with FDR *P*-value ≤ 0.05. X-axis values are average log2 (counts per million) and y-axis values are log2 (fold change). **(B)** Heatmap and hierarchical clustering of DE miRNAs.

**Figure 6:**
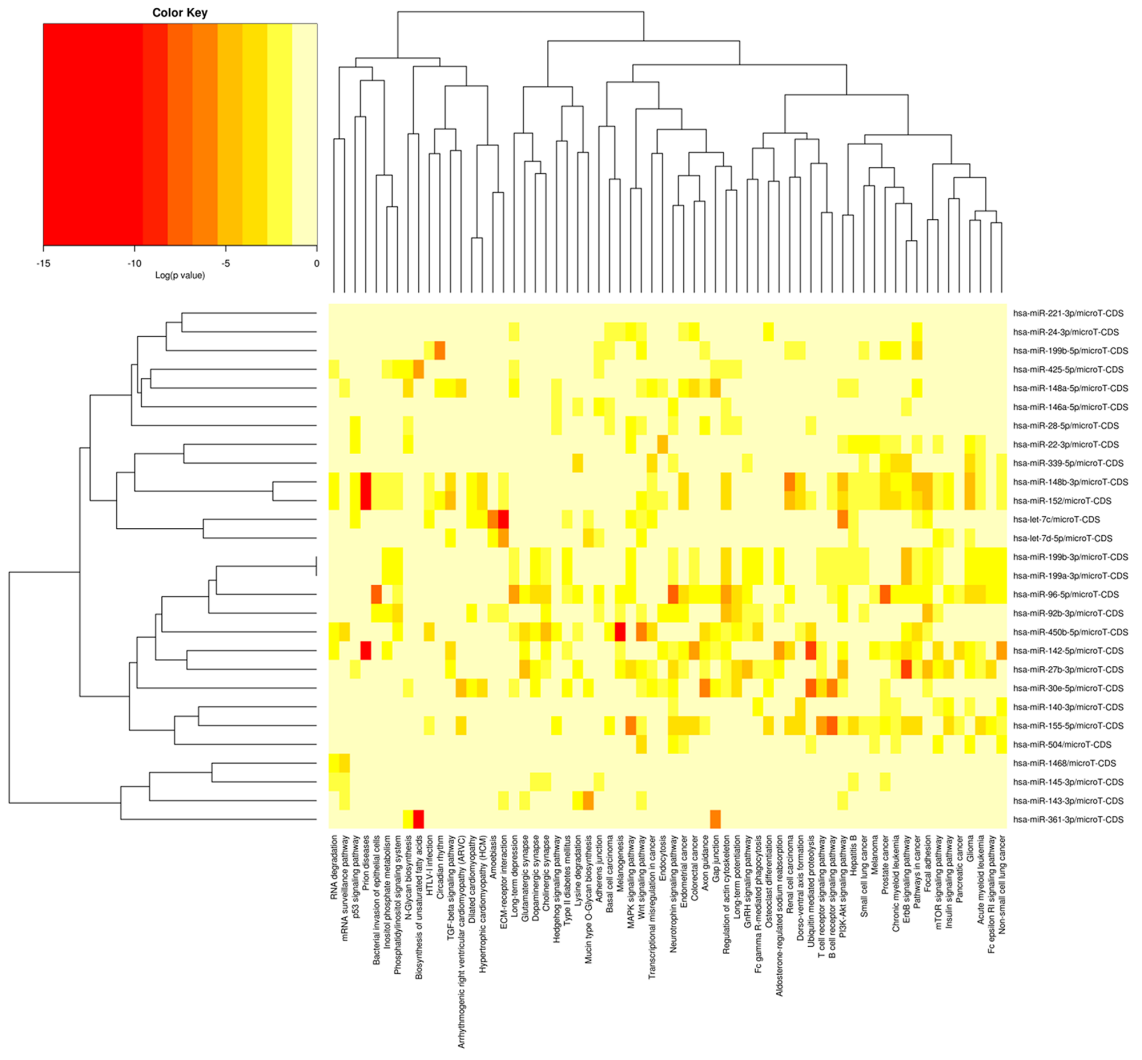
Heatmap and cluster patterns of the DE miRNAs and target-gene related pathways miRNAs are clustered together by exhibiting similar pathway targeting patterns, and pathways are clustered together by related miRNAs. As porcine genes were not included in the current version of DIANA miRPath, prediction was performed using human miRNAs.

A total of 58 known miRNAs were differentially expressed with a criterion of an FDR-value less than 0.05. Of these, 26 were upregulated and 32 were down-regulated in the H group relative to the L group. The differential expression of the 58 miRNAs in the H and L groups are presented in Figure 5. KEGG pathway analysis was performed to better understand the function of the DE miRNAs, and DIANA mirPath indicated these DE miRNAs were significantly enriched in the PI3K-Akt signaling pathway (p = 1.95E-36), the Wnt signaling pathway (p = 3.79E-35), and axon guidance (p = 3.44E-33). To further classify and predict the function of the DE miRNAs, we performed hierarchical clustering of the DE miRNAs and their target pathways (Figure 6). Some miRNAs, which were involved in similar regulation patterns or pathway functions, were clustered together, such as miR-148b-3p and miR-152.

### mRNA-miRNA regulatory network analysis

**Figure 7:**
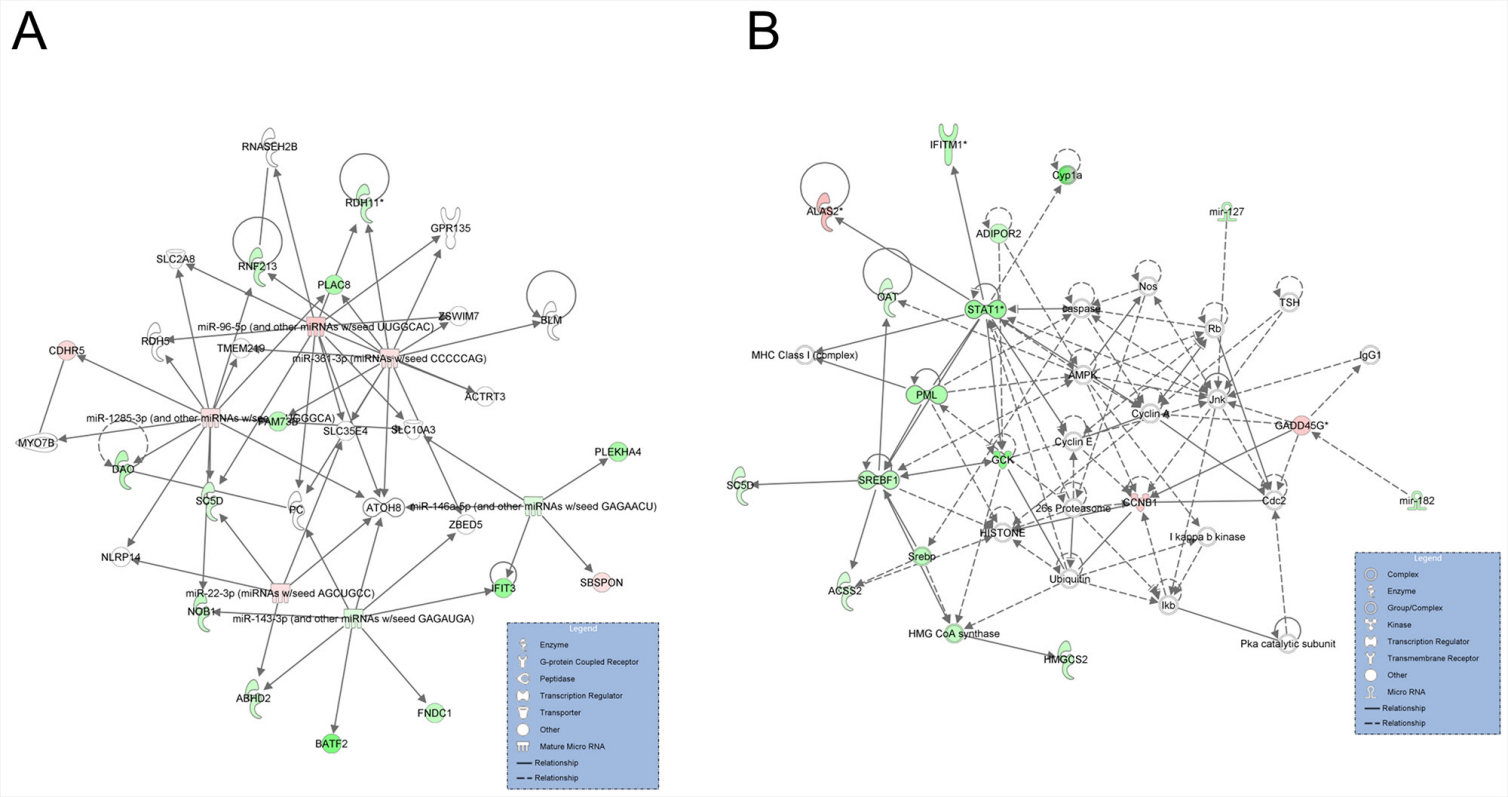
Graphical representation networks generated by IPA related to lipid metabolism **(A)** Network 1 (direct, score 32). **(B)** Network 2 (direct, score 23). Node shapes indicate the biological function of the genes and gene products, and the relationship among these is represented as a line. Node colors indicate gene expression: green indicate down-regulated and red indicates upregulated expression when comparing the H to L groups.

We constructed miRNA-mRNA interaction net-works based on the DEGs and DE miRNAs of the two groups using Ingenuity Pathway Analysis (IPA) software. Two networks, which were associated with lipid metabolism, were identified (Figure 7). The first network, scoring 32, contained 14 DEGs and 6 DE miRNAs (Figure 7A). Importantly, miR-146a-5p was one of the central molecules, which could target the *SBSPON* gene and repress its expression in the network. A total of 18 DEGs and 2 DE miRNAs were involved in the second network, which had a network score of 23. Most of the DEGs in the second network were upregulated in the H group compared with the L group, while *GADD45G* was down-regulated in the H group and could be targeted by miR-182 (Figure 7B).

### Validation of DEGs and DE miRNAs by RT-qPCR

**Figure 8:**
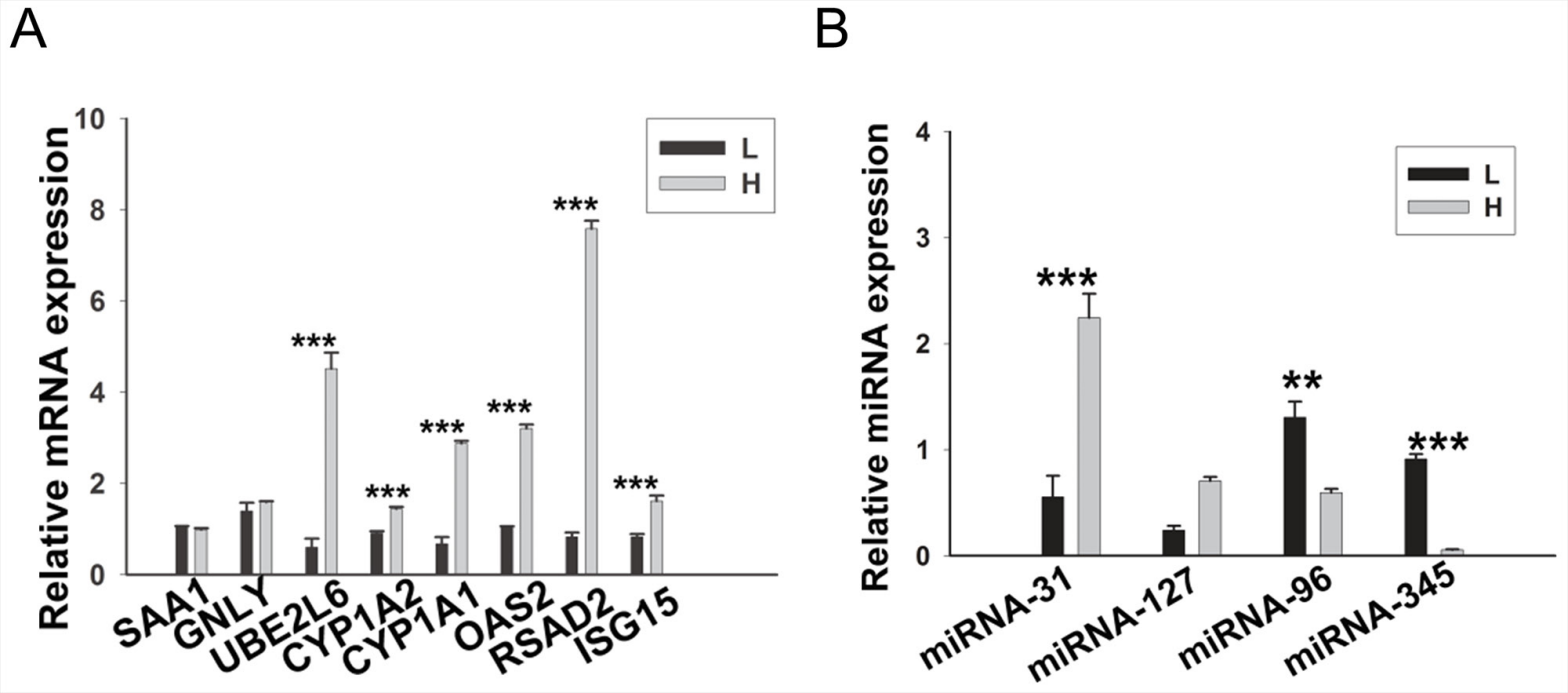
RT-qPCR validation of the DEGs and DE miRNAs **(A)** 8 DEGs were validated by RT-qPCR. **(B)** 4 DE miRNAs were validated by RT-qPCR. Porcine β-actin and U6 were selected as internal controls for mRNA and miRNA, respectively. This experiment was repeated three times. Data are shown as means ± S.E.M; ^**^P < 0.01 and ^***^P < 0.001.

Because no biological replicates were performed in our RNA-seq and miRNA-seq analysis and in order to verify the foregoing analyses, 8 DEGs and 4 DE miRNAs involved in lipid metabolism were selected and examined by RT-qPCR using three additional individuals from each group (Figure 8A-8B). The results of RT-qPCR were in good agreement with our sequencing data, indicating the reliability of our RNA-seq and miRNA-seq data.

## DISCUSSION

Growth rate and meat quality are two of the most important economic traits in pig production and breeding, and different selection strategies during pig breeding lead to significantly differences in fat content among pigs. Modern commercial pig breeds (Landrace, Yorkshire, and Duroc) have considerably higher growth rates and lower fat content than Chinese indigenous obese-type breeds (such as Yimeng black and Tongcheng pigs). With the improvement of people’s living standard, meat quality has become one of the more important indicators of consumer concerns in China. The current breeding goal for pigs in China is to seek a balance between growth rate and meat quality. Backfat thickness and intramuscular fat have a high positive correlation with meat quality in pigs; however, the genetics underlying fat deposition is complex and still largely unclear. In this study, high-throughput sequencing technologies were used to identify candidate genes and networks related to fat deposition, and the liver transcriptome profiles of mRNA and miRNAs in Yimeng Black pigs with extreme phenotypes in backfat thickness were presented. Approximately 59.75% to 60.40% of the RNA-seq and 82.3% to 83.5% of the miRNA-seq were mapped to the *Sus scrofa* reference genome.

We identified 238 DEGs between pigs with extreme high and low backfat thickness levels. Various genes associated with lipid metabolism demonstrated upregulated expression in the H group, such as *CYP1A1*, *CYP1A2*, *HMGCS2, ACSS2,* and *UBE2L6*. Cytochrome P450 family 1 subfamily member 1 and 2 (*CYP1A1* and *CYP1A2*) are involved in the oxidation of unsaturated fatty acids [29], and the upregulation of *CYP1A1* and *CYP1A2* in the H group was consistent with the observations of a previous study [24]. 3-hydroxy-3-methylglutaryl-CoA synthase 2 (*HMGCS2*) regulates mitochondrial fatty acid oxidation in liver [30], and acyl-CoA synthetase short-chain family member 2 (*ACSS2*) gene catalyzes the ATP-dependent activation of acetate and is involved in *de novo* lipid synthesis and energy generation [31]. Knockdown of ubiquitin conjugating enzyme E2 L6 (*UBE2L6*) in 3T3-L1 adipocytes can repress adipogenesis [32]. Upregulated expression of these genes in the H group indicated that lipid metabolism was likely upregulated in pigs with high backfat thickness compared to pigs with low backfat thickness.

miRNAs are small endogenous non-coding RNA with regulatory functions in eukaryotes, and approximately 40 miRNAs associated with adipogenesis have been recognized [33]. Various DE miRNAs associated with adipogenesis were identified in our study. Among them, miR-27a is a known suppressor of adipocyte differentiation via suppression of *PPARγ* expression, which is a master transcription factor for adipocyte differentiation [15, 18] and is a regulator of porcine adipocyte lipid metabolism [34]. miR-31 controls adipogenesis and regulates adipocyte differentiation by targeting the angiotensinogen (*AGT*) gene, which also plays an important role in adipocyte differentiation [35, 36]. However, the expression of miR-27a and miR-31 was higher in the H group compared to the L group. miR-34a is an inhibitor of beige and brown fat formation that functions by suppressing adipocyte *FGF21* signaling and *SIRT1* function [37], and our observations indicated that miR-34a was down-regulated in the H group.

Functional annotation results showed that the DEGs involved in oxidative stress were significantly enriched, such as *OLR1*, *PHYHD1*, and *DUSP1*. It’s reported that oxidative stress in the liver modulates lipid metabolism and contributes to fat deposition [38]. Oxidized low-density lipoprotein receptor 1 (*OLR1*) mediates the recognition, internalization, and degradation of oxidatively modified low-density lipoprotein, which is associated with cell migration, proliferation, inhibition of apoptosis, and lipogenesis [39]. Phytanoyl-CoA dioxygenase domain containing 1 (*PHYHD1*) is highly conserved in eukaryotes and is involved in the oxidation of Fe (II) and might catalyze oxidation the fatty acid-CoA esters [40]. Dual specificity phosphatase 1 (*DUSP1*) is a potential inhibitor of MAPK activity, and *DUSP1* is a transcriptional target of p53 involved in signaling apoptosis after oxidative stress [41]. We proposed that these genes associated with oxidative stress might be responsible for fat deposition in pigs.

The miRNA-mRNA regulatory networks highlighted in this study have provided a comprehensive profile for understanding the mechanism of fat deposition in pigs. Two miRNA-mRNA interaction networks associated with lipid metabolism were identified. miR-143 and miR-146a were the hub molecules in network 1, while miR-143 and miR-146a-5p were upregulated in the livers of the H group. It has been reported that miR-143 regulates adipocyte differentiation [42] and has a certain role in regulating insulin resistance [43]. Moreover, miR-143 can regulate lipid metabolism in porcine adipocytes [44]. miR-146a-5p inhibits adipogenesis by suppressing insulin resistance protein expression in primary porcine adipocytes [45]. Signal transducer and activator of transcription 1 (*STAT1*) and sterol regulatory element binding transcription factor 1 (*SREBF1*) were the central molecules in network 2 and were upregulated in the H group. *STAT1*, a downstream effector common to both IFN-α and IFN-γ, plays a prominent role in apoptosis, immunity, and lipid metabolism [46]. *SREBF1* plays a critical role in promoting *in vitro* adipocyte differentiation and regulation of lipid homeostasis [47, 48]. These findings indicated that the expression change in DEGs and DE miRNAs within these two networks might contribute to lipid metabolism and fat deposition in pigs.

## MATERIALS AND METHODS

### Experimental design, animals, and phenotypes

The Yimeng black pig population (8 months old; average live weight 100 kg; range, 99-105 kg) had access to the same food three times a day and water *ad libitum* under the same management conditions. The average live backfat thickness of Yimeng black pigs was measured at the three positions, including posterior edge of shoulder, final rib, and lumbosacral junction. A total of 6 pigs with high backfat thickness (H group) and 6 pigs with low backfat thickness (L group) were selected based on their pedigrees, and full/half-sibs were selected to minimize the noise of different genetic backgrounds. The chosen pigs were slaughtered according to the guidelines defined by national and local animal welfare and approved by the Institutional Animal Care and Use Committee at Shandong Agricultural University.

After slaughter, the production and carcass traits were recorded for all 12 individuals. Two pigs in each group, both of which were full-sibs, were selected to perform RNA-seq and miR-seq analyses. These four pigs had no differences in body weight, carcass weight, leaf lard weight, or lean percentage. The liver tissues of these four pigs were collected after slaughter and frozen in liquid nitrogen until RNA extraction.

### mRNA sequencing and data analysis

Total RNA was isolated using Trizol Reagent (Invitrogen, Carlsbad, CA, USA) following the manufacturer’s protocol. The quality and concentration of total RNA was detected using a RNA 6000 Nano LabChip Kit and a 2100 Bioanalyzer (Agilent Technologies, Santa Clara, CA, USA). Two mRNA libraries were constructed for each group (H and L) from total RNAs pooled from two individuals. Poly (A) mRNA was isolated from total RNA with poly-T oligo attached magnetic beads (Invitrogen, Carlsbad, CA, USA). Following purification, the purified mRNA was randomly fragmented using divalent cations under elevated temperature. Then, the cleaved RNA fragments were reverse-transcribed to create the final cDNA library in accordance with the protocol for the mRNA-Seq sample preparation kit (Illumina, San Diego, CA, USA), and the average insert size for the single end libraries was 300 bp (±50 bp). Then, single-end libraries (H and L groups) were sequenced using an Illumina GAIIx sequencing system according to the manufacturer’s instructions, which were performed at LC-Bio Co. (Hangzhou, China).

FastQC software was used to evaluate the whole quality of sequencing data (http://www.bioinformatics.babraham.ac.uk/projects/fastqc/). Clean reads were obtained by filtering adaptor sequences and removing low-quality sequences. The clean reads were then mapped to the reference genome of pig (*Sus scrofa* build 10.2) [49] using Bowtie software (version 2.1.0) with default parameters [50]. Only reads with a perfect match or 1 mismatch were further considered and annotated. The expression level of each gene was estimated as number of reads per kilobase per million reads (RPKM) [51]. DEGs were identified with the criteria of |fold change| ≥ 1.5 and false discovery rate (FDR) ≤ 0.05 using the edgeR package [52] in R software (http://cran.r-projects.org).

### Small RNA sequencing and data analysis

We performed sRNA sequencing of the same samples as transcriptome sequencing. Approximately 1μg of total RNA was used to prepare sRNA libraries according to protocol of TruSeq^TM^ Small RNA Sample Prep Kits (Illumina, San Diego, CA, USA). The libraries were directly submitted for 36bp single-end sequencing using an Illumina Hiseq 2000 sequencing platform according to the manufacturer’s instructions at the LC-BIO Co. (Hangzhou, China). The adapters of the raw data were removed using cutadapt (v 1.10) [53], and the reads shorter than 18 bp or longer than 32 bp were then discarded using an in-house Perl script. The clean reads were aligned to the *Sus scrofa* reference genome (build 10.2) using miRDeep2 software (version 2.0.0.8) [54]. The sequences of mature miRNAs and their precursors were downloaded from the miRBase database (http://www.mirbase.org/) [55]. Prediction of novel miRNAs and calculation of read counts for each miRNA were performed using miRDeep2 software. The expression level of each miRNA was normalized using the transcripts per kilobase million (TPM) method. EdgeR package [52] in R (http://cran.r-projects.org) was used to identify DE miRNA between the H and L group. miRNAs with FDR adjusted *P*-value ≤ 0.05 were considered to be differentially expressed.

### Bioinformatics analysis

GO and KEGG pathway enrichment were performed using the Database for Annotation, Visualization, and Integrated Discovery (DAVID) website (http://david.abcc.ncifcrf.gov/) [56]. The EASE value was set to 0.05 for the enrichment analysis. To explore the potential function of miRNAs with significantly differential expression between the two groups, potential target genes and pathways of miRNAs were predicted by DIANA miR Path (v.2.0) (http://www.microrna.gr/miRPathv2) using a *P*-value threshold of 0.05 and a MicroT threshold of 0.860. As porcine genes were not included in the current version of DIANA miR Path, pig miRNAs IDs were converted into human miRNAs IDs before predicting their targets. The miRNA-mRNA interaction networks were constructed using Ingenuity Pathways Analysis (IPA) software (Qiagen, Valencia, CA, USA) with a cutoff of 35 molecules per network and 25 networks per analysis.

### Real-time quantitative PCR

The isolated RNA of individual samples was reverse transcribed into cDNA using a RevertAid First-Strand cDNA Synthesis Kit (Thermo, Waltham, MA, USA) in a total volume of 25 μL containing 2 μg of total RNA, following the manufacturer’s instructions. A total of 2 μg of miRNA was reverse-transcribed using 0.5 μL specific primers and 0.5 μL *U6* antisense primers, with other conditions remaining unchanged. PCR primers were designed using Primer Premier 5 software and are shown in Table 2 and Table 3. The mRNA reaction solution was comprised of 10 μL of 2× SYBR^®^ Premix Ex Taq, 0.4 μL of each primer, 1 μL of cDNA, 0.4 μL of Dye II, and sterile water to a volume 20 μL. The miRNA reaction solution was comprised of 10 μL of 2× SYBR^®^ Premix Ex Taq II, with the other components unchanged. The PCR program was performed at 95°C for 5 min, followed by 40 cycles at 95°C for 5 s and 60°C for 1 min. Thereafter, a dissociation program was carried out at 95°C for 15 s, 60°C for 1 min, and 95°C for 15 s. Each reaction was performed in triplicate. The 2^−ΔΔCt^ method was used to determine the gene expression level [57]. Porcine *β-actin* and *U6* genes were selected as internal controls for mRNA and miRNA, respectively [58]. A t-test was used to evaluate the expression difference.

**Table 2:** Primers designed for RT-PCR and RT-qPCR to validate differentially expressed miRNAs

Primer name	Primer sequence (5′→3′)	Use
miR-96	GTCGTATCCAGTGCAGGGTCCGAGGT	RT-PCR
	ATTCGCACTGGATACGACAAACGA	
miR-345	GTCGTATCCAGTGCAGGGTCCGAGGT	RT-PCR
	ATTCGCACTGGATACGACGCACTG	
miR-127	GTCGTATCCAGTGCAGGGTCCGAGGT	RT-PCR
	ATTCGCACTGGATACGACAGCCAA	
miR-31	GTCGTATCCAGTGCAGGGTCCGAGGT	RT-PCR
	ATTCGCACTGGATACGACCAGCTA	
miR-96	TTTGGCACTAGCACATTCCG	RT-qPCR
miR-345	GCTGACTCCTAGTCGCGC	RT-qPCR
miR-127	TCGGATCCGTCTGAGCC	RT-qPCR
miR-31	AGGCAAGATGCTGGCACC	RT-qPCR
universal AP	CTCAACTGGTGTCGTGGAGTC	RT-qPCR
U6	F: GCTTCGGCAGCACATATACTAAAAT	
	R: CGCTTCACGAATTTGCGTGTCAT	

**Table 3:** Primers designed for the validation of differentially expressed genes by RT-qPCR

Primer name	Primer sequence (5′→3′)
*GNLY*	F: GCTGTCCTGCTCCTCACCTT
	R:GCAGAGTGCTCAGGGGTCAG
*UBE2L6*	F: TCAGAAGGAGCTTCCCAGGT
	R: GGTTGTAGGGCGGTTTCTCA
*CYP1A1*	F: CGCTATGGAGATGTGCTGC
	R: CCGTCCCTTGAAATCGTC
*CYP1A2*	F: CTCCTTCGTTCCCTTCACC
	R: GCCACTGGTTTACGAGGAC
*ISG15*	F: TCTAGAAATGCCCCCTTGCC
	R: GCAAAAGCTCCTAGAGCCCA
*OAS2*	F: TCGTCGTGTTCACAGACTTGC
	R: CCTGGGAGCCTTCCATTTT
*RSAD2*	F: GGGAGAGGTGGTTCAAGAGC
	R: GACCACGGCCAATAAGGACA
*SAA1*	F: GGTGCCTGGGCTGCTAA
	R: CAGGAGGTCTGAAGTGGTTGG
*β-actin*	F: CAAGGCCAACCGTGAGAAGA
	R: TTCTCCTTGATGTCCCGCAC

## CONCLUSIONS

We used mRNA and miRNA transcriptomes to explore the liver transcriptome of pigs with different backfat thicknesses, and a total of 238 DEGs and 58 DE miRNAs were identified. A number of genes and miRNAs involved in lipid metabolism were differentially expressed, which agreed with the phenotypic differences between the H and L group in regard to fatness traits. Additionally, based on functional enrichment and miRNA-mRNA interaction analysis, the potential roles of lipid metabolism and oxidative stress in regulating fat deposition were highlighted. Overall, this study provided new candidate genes and miRNAs associated with fat deposition. Our findings will be of use in selection strategies to improve the quality of pork meat and combating obesity in humans. Moreover, it is worth noting that the exact biological role of the candidate genes and miRNAs involved in fat deposition still require further characterization by way of functional studies.

## Abbreviations

DAVID: Database for Annotation, Visualization, and Integrated Discovery
DE: differentially expressed
DEG: differentially expressed gene
FC: fold change
FDR: false discovery rate
GO: gene ontology
IR: insulin resistance
IMF: intramuscular fat
IPA: ingenuity pathway analysis
KEGG: Kyoto Encyclopedia of Genes and Genomes
microRNA: miRNA
RPKM: reads per kilobases per million reads
RT-PCR: reverse transcription polymerase chain reaction
RT-qPCR: real time quantitative polymerase chain reaction
SAT: subcutaneous adipose tissue
sRNA: small RNA
